# Contributions of expected learning progress and perceptual novelty to curiosity-driven exploration

**DOI:** 10.1016/j.cognition.2022.105119

**Published:** 2022-08

**Authors:** Francesco Poli, Marlene Meyer, Rogier B. Mars, Sabine Hunnius

**Affiliations:** a Donders Institute for Brain, Cognition and Behaviour, Radboud University, Nijmegen, the Netherlands; b Wellcome Centre for Integrative Neuroimaging, Centre for Functional MRI of the Brain (FMRIB), Nuffield Department of Clinical Neurosciences, John Radcliffe Hospital, University of Oxford, Oxford, UK

**Keywords:** Curiosity, Novelty, Exploration, Learning progress, Uncertainty, Computational modeling

## Abstract

Exploration is curiosity-driven when it relies on the intrinsic motivation to know rather than on extrinsic rewards. Recent evidence shows that artificial agents perform better on a variety of tasks when their learning is curiosity-driven, and humans often engage in curiosity-driven learning when sampling information from the environment. However, which mechanisms underlie curiosity is still unclear. Here, we let participants freely explore different unknown environments that contained learnable sequences of events with varying degrees of noise and volatility. A hierarchical reinforcement learning model captured how participants were learning in these different kinds of unknown environments, and it also tracked the errors they expected to make and the learning opportunities they were planning to seek. With this computational approach, we show that participants' exploratory behavior is guided by learning progress and perceptual novelty. Moreover, we demonstrate an overall tendency of participants to avoid extreme forms of uncertainty. These findings elucidate the cognitive mechanisms that underlie curiosity-driven exploration of unknown environments. Implications of this novel way of quantifying curiosity within a reinforcement learning framework are discussed.

## 1 Introduction

The brain is often described as a prediction engine (Clark, 2013). As such, it works constantly to maximize its predictive power over the environment. This allows us to act effectively in the world around us. It is relatively easy to accomplish successful actions when we move in familiar environments in which we have all the information we need to make optimal decisions; how we sample information in such known environments has been studied thoroughly (Kolling et al., 2016; Kolling, Behrens, Mars, & Rushworth, 2012; Shenhav, Straccia, Botvinick, & Cohen, 2016; Tomov, Truong, Hundia, & Gershman, 2020). However, to devise an efficient information search in unknown environments is much harder (Baranes, Oudeyer, & Gottlieb, 2014).

Recently, the field of artificial intelligence has made great progress towards the solution of this puzzle (Bellemare et al., 2016). The turning point has been the development of artificial agents whose exploratory behavior is driven by curiosity rather than external rewards. The key idea here is that when external rewards are sparse or unknown, agents are better off relying on an intrinsic drive to get to know what is unknown (Pathak, Agrawal, Efros, & Darrell, 2017). Yet, curiosity is a broad concept, and different factors might underlie it. In other words, the specific utility function that agents are aiming to maximize when exploring the environment can take several different forms (Ten, Oudeyer, & Moulin-Frier, 2021). Information gain (Ruggeri, Pelz, & Schulz, 2021), the utility of knowledge (Dubey & Griffiths, 2020), learning progress (Oudeyer, 2007), and novelty (Berlyne, 1950; Smock & Holt, 1962) are only some of the factors that have been proposed to underlie curiosity both in natural and artificial agents.

The maximization of learning progress relies on the idea that agents find improving their performance rewarding in itself (Burda et al., 2019; Houthooft et al., 2016). As a consequence, agents explore the world around them with the goal to maximize their learning (Baldassarre et al., 2014; Oudeyer, 2007). Evidence on whether human curiosity-driven exploration is based on the maximization of learning progress is inconsistent. A number of recent developmental studies showed that, from a very young age, we tailor our attention to maximize the information we gather from the environment (Addyman & Mareschal, 2013; Liquin, Callaway, & Lombrozo, 2021; Poli, Serino, Mars, & Hunnius, 2020). For example, we showed that when infants were presented with unknown (yet learnable) sequences of probabilistic events, they were likely to keep looking at the sequence as long as the information gain was high, but tended to direct their attention away if the stimuli did not offer a learning opportunity (Poli et al., 2020). Hence, humans are sensitive to the informational structure of the environment from early on, but current evidence falls short in demonstrating whether we make use of this sensitivity to actively structure our exploration in unknown environments.

In contrast, studies on adults have to date failed to find evidence for an information search strategy that relies on learning progress (Baranes et al., 2014; Wade & Kidd, 2019). Baranes et al. (2014) tested adults in an exploration game in which 64 games of different difficulty were available, and participants could choose which one to engage with and for how long. They were also asked to report how much they improved in the game they picked (i.e., their learning progress) and how much they expected to improve in the future (i.e., the expected learning progress). If participants' exploration is driven by maximizing learning progress, they should keep focusing on the same game as long as learning progress is substantial, but switch to another game if progress is absent or minimal. However, the results by Baranes et al. (2014) showed that self-reports of learning progress were unrelated to future choices. Rather, participants' choices depended on game difficulty and novelty: they tended to go from easier to harder games, with a general preference for novel games over familiar ones. These findings open up the possibility that, when humans are faced with open-ended and unknown environments, information search is not aimed to maximize learning progress, but rather novelty-based or even random (see also Schwartenbeck et al., 2019). However, the study by Baranes et al. (2014) relied on self-reports, which can be a rather imprecise measure of learning progress as they require good self-monitoring skills (Schwarz & Oyserman, 2001) and might reflect a conscious process potentially different from that observed in artificial agents altogether.

Novelty seeking has been suggested to guide exploration in unknown environments as well. There is a long tradition of research on the role of novelty in curiosity-driven learning (Berlyne, 1950; Smock & Holt, 1962). In this work, novelty is usually defined as a multi-dimensional construct that is divided in an epistemic and a perceptual component (Berlyne, 1950), both of which have been found to activate reward networks in the brain, favoring the idea that novelty is rewarding in itself (Wittmann, Bunzeck, Dolan, & Düzel, 2007; Wittmann, Daw, Seymour, & Dolan, 2008). However, even if recent work on reinforcement learning shows the efficiency of novelty-based strategies in exploration tasks (Lehman & Stanley, 2011; Tang et al., 2017), novelty alone cannot explain human exploration in unknown environments, because novelty-based exploration can push agents into unlearnable situations that are devoid of useful information (Gottlieb, Oudeyer, Lopes, & Baranes, 2013).

In the current study, we adopted a novel paradigm that combines participants' exploration with minimal task instructions and no external rewards. This gave us the possibility to create unknown environments that are optimal to investigate curiosity-driven exploration and its underlying cognitive mechanisms. Specifically, we manipulated different kinds of environmental uncertainty (Nassar, McGuire, Ritz, & Kable, 2019; O'Reilly, 2013) to create experimental situations that triggered curiosity. A first kind of uncertainty is noise, which consists of irregular fluctuations that affect a stimulus but are not part of it and tend to obscure it. Another kind of uncertainty is volatility, which entails how quickly the stimulus or environment can change. Both noise and volatility make a stimulus unreliable, thus creating uncertainty. It has been studied in great detail how the human brain processes noise and volatility (Findling, Skvortsova, Dromnelle, Palminteri, & Wyart, 2019; Gómez, 2002; Nassar, Bruckner, & Frank, 2019), so we used them here as a tool to investigate curiosity-driven exploration (see also Stojić, Schulz, Analytis, & P., & Speekenbrink, M., 2020). Specifically, introducing uncertainty allowed us to generate unknown (yet learnable) environments where we could test whether exploration was based on learning progress maximization, perceptual novelty, or whether it was random instead. If exploration is driven by learning progress, participants are expected to disengage from a stimulus when the stimulus does not offer a learning opportunity. Moreover, they should preferentially explore the stimulus that offers the greatest expected learning progress. Alternatively, participants might prefer stimuli that are more novel (i.e., novelty-based exploration) or switch randomly between stimuli (random exploration). We employed computational modeling to quantify these factors. Hence, differently from the self-report tests administered in previous studies, our approach allows for a precise quantification of learning progress without need for conscious verbal report.

Importantly, our study departs from literature on the exploitation-exploration trade-off, as it does not use extrinsic rewards to study the balance between explorative and exploitative behavior (for a review, see Wilson, Bonawitz, Costa, & Ebitz, 2021). As a consequence, we cannot evaluate participants' performance in terms of their ability to maximize rewards. However, we could track their *predictive* performance. In other words, we tested whether the curiosity-driven exploratory strategies that participants adopted were successfully minimizing prediction errors. Moreover, we investigated whether their predictive performance was stable across different kinds of uncertainty, and whether they adapted their learning depending on the specific environment they were exploring.

## 2 Methods

### 2.1 Participants

Sixty-seven participants were recruited via an online participant management software called Sona (sona-systems.com) and received university credits for their participation. Nine participants were discarded from the analysis because they did not meet the inclusion criteria (see below), and data of three additional participants were not collected due to technical problems. The final sample consisted of 55 participants (M_age_ = 20.44, *SD* = 2.85, F = 28, M = 27). The study was approved by the faculty's ethics committee.

### 2.2 Procedure

Participants were tested online. The experimenter instructed participants via Zoom (zoom.us) on how to open the online game. The game was coded in PsychoPy 3.2 (Peirce et al., 2019) and then uploaded on Pavlovia (pavlovia.org). Participants played while the Zoom call was open, in case any problem arose during the experiment. The Zoom session was not recorded. After the online game, participants reported which of the characters they played with during the game was their favorite, which was their least favorite, and whether they followed the instructions during the task.

### 2.3 Materials

**Fig. 1:**
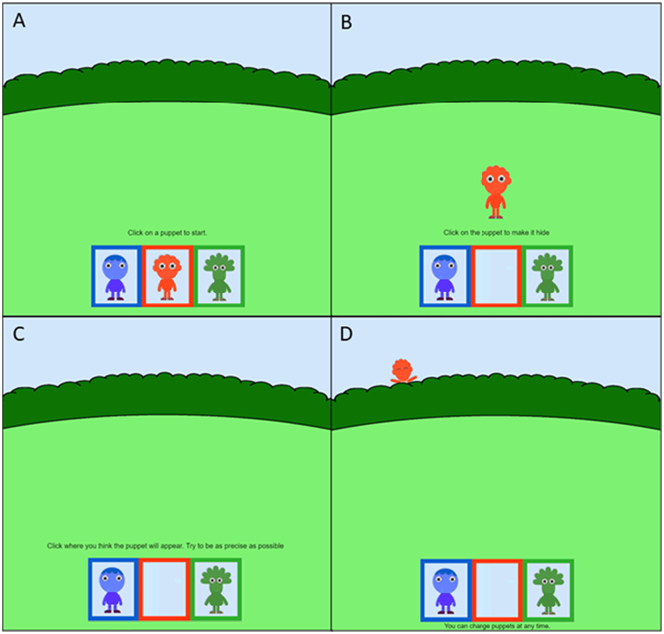
Participants started the task by choosing which character to play with initially (A) and had to click on it to make it hide behind the hedge (B). Then, they had to guess where the character would reappear (C) and received feedback on its actual location (D). Instructions were present only during the first trial. Note: Characters are blurred to avoid copyright infringement.

Participants were presented with three characters that were distinguishable by their color (green, blue, and red) and shape (Fig. 1A). To start, participants were asked to pick one of the characters. The selected character appeared in the middle of the screen, below a long hedge (Fig. 1B). When participants clicked on the character, it hid behind the hedge. Participants had to click on the hedge, trying to guess where the character would reappear (Fig. 1C). Upon their button click, the character reappeared from behind the hedge (Fig. 1D). The other characters remained visible on the screen, and participants were free to decide whether to keep playing with the same character or switch to a different one at any moment throughout the game. They received no instructions on where the characters would hide or on how to find them. Also, they received no external rewards for correctly guessing the character's hiding spot.

**Fig. 2:**
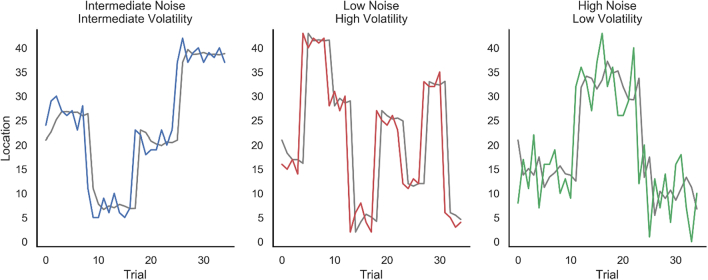
The hiding patterns of the three characters across time (in colours) and the performance of the ideal learner model (in grey) in predicting their location. On the y-axis, zero indicates the far left of the screen. (For interpretation of the references to color in this figure legend, the reader is referred to the web version of this article.)

The three characters were hiding following a Gaussian distribution. Two parameters of the Gaussian distribution (mean and standard deviation) were manipulated independently for each character, so that the three characters followed three different hiding patterns, as depicted in Fig. 2. Specifically, the characters had three different levels of noise (i.e., the standard deviation of the gaussian distribution) and three different levels of volatility (i.e., how often the mean of the distribution changed). A first character had high noise and low volatility, another had low noise and high volatility, and a third had intermediate noise and volatility. Which character (blue, green, or red) belonged to each hiding pattern was counterbalanced across participants. After any of the characters had hidden 35 times, the game ended. Hence, the overall number of trials varied between participants (M = 74, *SD* = 13, range = 38–85).

### 2.4 Computational modeling

We implemented a reduced Bayesian learner (inspired by Nassar, Wilson, Heasly, & Gold, 2010) that acquires and updates evidence in an optimal way. This implies inferring the mean and the standard deviation of hidden probability distributions relying on previous observations, i.e., *p*(*X*_*t*+1_| *X*_1:*t*_), as well as detecting change-points (i.e., when the mean of the distribution changes). Hence, after sampling a new piece of evidence, the model computes how likely it is that a change-point *cp* occurred. The change-point probability is computed using Bayes' theorem:

(1) $$p\left(\mathrm{cp}|X_{t}\right)=\frac{U(X_{t}\left|\mathrm{range}\right)H}{U(X_{t}\left|\mathrm{range}\right)H+N\left({\hat{\mu }}_{t},{\hat{\sigma }}_{t}\right)\left(1-H\right)}$$

where U and N indicate a uniform and a normal distribution, respectively. Hence, the hidden probability distribution of a given character is uniform over the entire space if a change-point has occurred, and it is normal with mean ${\hat{\mu }}_{t}$ and standard deviation ${\hat{\sigma }}_{t}$ if a change-point has not occurred. *X* is a vector containing all the evidence that has been observed and *X*_*t*_ is the last piece of evidence that has been observed; *range* is a vector containing any location that the character can take; H is a constant that specifies the average hazard rate of any given sequence (scripts for details are available at osf.io/tqevz/). In other words, it specifies the rate of change of the distribution mean of a character (the higher the hazard rate, the more frequently the mean changes). It is important to notice that the change-point probability is used by the model to detect a change-point after it happened, while change-points cannot be predicted before they happen.

The expected mean and standard deviation of the distribution are updated trial by trial, and both depend on whether a change-point has been estimated to occur or not. When estimating the mean, if a change-point occurred, only the last piece of evidence that has been observed should be relevant:

(2) $${\hat{\mu }}_{t}^{\mathrm{cp}}=X_{t}$$

Hence, all the evidence up until trial t-1 will be disregarded. Conversely, also older evidence is taken into account if a change-point has not occurred:

(3) $${\hat{\mu }}_{t}^{\neg \mathrm{cp}}=\frac{X_{t}+{\hat{r}}_{t}{\hat{\mu }}_{t-1}\;}{{\hat{r}}_{t}+1}$$

where the expected run length ${\hat{r}}_{t}$ indicates the expected number of consecutive trials (i.e., a run) since the last change-point. In words, Eq. (3) integrates the evidence acquired in the last trial with the expectations that the model had up until the previous point in time, weighting them for the time that passed from the last change point. The final estimate of the mean keeps into account both the possibility that a change point occurred and the possibility that it did not, weighting them for the probability that a change-point has actually occurred:

(4) $${\hat{\mu }}_{t}={\hat{\mu }}_{t}^{\mathrm{cp}}p\left(\mathrm{cp}|X_{t}\right)+{\hat{\mu }}_{t}^{\neg \mathrm{cp}}\left(1-p\left(\mathrm{cp}|X_{t}\right)\right)$$

Since inferring the current mean is a weighted integration of past expectations and new evidence, it can be re-written as a reinforcement learning algorithm (often called Rescorla-Wagner rule):

(5) $${\hat{\mu }}_{t}={\hat{\mu }}_{t}+\alpha_{t}\left(X_{t}-{\hat{\mu }}_{t}\right)$$

where *α*_*t*_ is the learning rate that regulates the extent to which the difference between previous expectations and new evidence (i.e., the prediction error) will change future expectations. The learning rate is computed as follows:

(6) $$\alpha_{t}=\frac{1+p\left(\mathrm{cp}|X_{t}\right){\hat{r}}_{t}}{{\hat{r}}_{t}+1}$$

In words, whether the prediction error will be considered informative depends on the probability that the current evidence indicates a change point, weighted by the expected run length ${\hat{r}}_{t}$.

Together with the estimation of the mean of the distribution, the model also estimates its standard deviation. The expected standard deviation of the probability distribution is updated adjusting the estimate of the previous trial given the new estimated mean, keeping into account the hazard rate and the change-point probability:

(7) $${\mathrm{SD}}_{t+1}^{2}={\mathrm{SD}}_{t}^{2}+\left(\frac{{\hat{r}}_{t}\left(X_{t}-{\hat{\mu }}_{t}\right)\;}{{\hat{r}}_{t}+1}-{\mathrm{SD}}_{t}^{2}\right)\mathrm{kH}\left(1-p\left(\mathrm{cp}|X_{t}\right)\right)$$

where k is a scaling constant. Hence, by keeping track of the change-point probability, we can estimate and update trial by trial the mean and the standard deviation of the hidden probability distributions of the three characters. From these estimates, we can compute the current and the expected learning progress. The current learning progress *LP*_*t*_ is computed as the difference in prediction error from the previous and the current trial:

(8) $${\mathrm{LP}}_{t}=\left(X_{t-1}-{\hat{\mu }}_{t-1}\right)-\left(X_{t}-{\hat{\mu }}_{t}\right)$$

This indicates how much the model has changed its prediction error during the last timepoint. Hence, it is a measure of how much the model has improved (or gotten worse). To estimate the expected learning progress *LP*_*expected*(*t*+1)_, a similar computation is carried out, but instead of using the prediction error from the past, the current prediction error is subtracted by the expected prediction error:

(9) $${\mathrm{LP}}_{\mathrm{expected}\left(t+1\right)}=\left(X_{t}-{\hat{\mu }}_{t}\right)-{\mathrm{PE}}_{\mathrm{expected}\left(t+1\right)}$$

where *PE*_*expected*(*t*+1)_ is the expected prediction error that the model estimates to make in the future. *PE*_*expected*(*t*+1)_ is estimated with a reinforcement learning algorithm:

(10) $${\mathrm{PE}}_{\mathrm{expected}\left(t+1\right)}={\mathrm{PE}}_{\mathrm{expected}\left(t\right)}+\alpha_{t}\left(\left(X_{t}-{\hat{\mu }}_{t}\right)-{\mathrm{PE}}_{\mathrm{expected}\left(t\right)}\right)$$

It is important to note that Eq. (5) was a reinforcement learning algorithm that was updating the expected mean, while this is a second reinforcement learning algorithm that relies on the first to update the expected prediction error. Hence, a hierarchical structure emerges, where the expected mean is estimated at the first layer of the reinforcement learning algorithm, and the prediction error is estimated at the second layer. Following Wittmann et al. (2016), we use the same learning rate *α*_*t*_ for both layers and, since the number of trials for each participant was low, *α*_*t*_ was estimated at the group level.

At any time-point, there is a fixed probability of switching environment just by chance. Hence, the cumulative probability of switching by chance increases as the true run length *r*_*t*_ increases, and drops when a change-point actually occurs. Random search (R) can be defined as a non-parametric function of the true run length *r*_*t*_:

(11) $$R=s\left(r_{t}\right)$$

where *s* is a smooth function, meaning that the relationship between R and *r*_*t*_ is specified by a function *s* that is estimated semi-parametrically. Finally, the more one is exposed to a given environment, the less novel it is. We quantified perceptual novelty (N) as a non-parametric function of negative overall exposure to a given character:

(12) $$N=s\left(-t\right)$$

where t indicates the overall number of trials a given character has been observed, and s is a smooth function. Given that we did not have a-priori assumptions on the rate of change in novelty or random search as a function of time, we used additive terms instead of, for instance, a logarithmic or exponential function. The additive term uses smooth functions to fit the data non-parametrically, thus allowing any nonlinear relation between data and regressors.

After generating the model, we tested whether we could successfully recover the learning rate *α* given the number of participants and trials in our data. We simulated data from 60 synthetic participants using fixed parameter values, and then fitted the data to the model. We found that the trial-by-trial estimates of the *α* parameter of the synthetic data were highly correlated with the *α* recovered by our model (*r* = 0.978), giving us evidence that we can successfully recover this parameter from the data. Then, we fitted the model to the participants' data with *α* as a free parameter and using a constrained-search algorithm that minimizes the squared difference between each participant and the model estimates. This allows us to infer the parameter values that better describe each individual's performance. Finally, we used the variables *LP*, *LP*_*expected*_, *N*, and *R* to predict when the participants switched environments and what environment they decided to sample next using generalized mixed-effects models.

### 2.5 Predictions

Computational modeling allowed the quantification of three different factors that could drive exploration (learning progress, perceptual novelty, and random search). Based on these three factors, we made distinct predictions on whether participants would continue sampling information from an environment or switch to a different one, and in the latter case, what environment they would choose to explore between the two viable options.

• Learning-maximization makes two specific predictions: participants' probability of switching to another stimulus depends on the learning progress, and the selection of the new stimulus depends on the difference in the expected learning progress between the two available options.
• Novelty-driven exploration assumes that participants will prefer the most novel stimulus over the others. We assumed the perceptual novelty of a stimulus to decrease as a function of time spent interacting with it. If exploration is driven by perceptual novelty, participants' probability of switching to another stimulus increases as stimulus novelty decreases. Moreover, the selection of the new environment to sample should depend on the difference in perceptual novelty between the available environments.
• Random search assumes that participants will disengage from a stimulus at any random point. Given that the probability of a random switch is fixed, participants' cumulative probability of switching to another stimulus is expected to increase as the time from the last switch increases. Regarding what environments participants will select next, random search does not make any specific prediction.

Given the aim of studying exploratory behavior, participants that did not explore more than one environment (*N* = 4) were discarded from the analysis. Additionally, participants who reported to have performed the task without following the instructions were discarded from the analysis (*N* = 5).

## 3 Results

### 3.1 Exploration termination

**Fig. 3:**
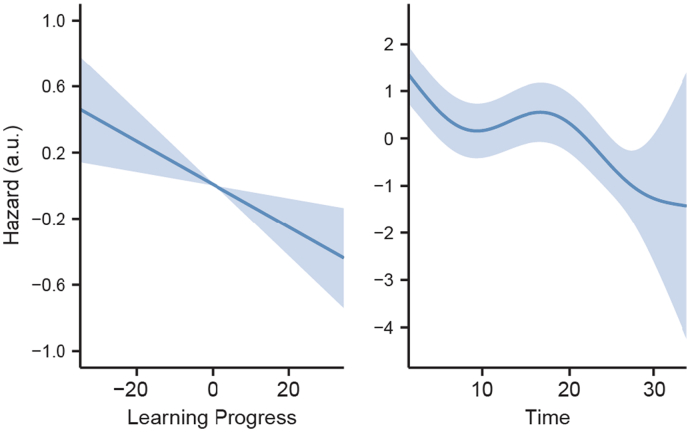
Analysis of the probability of switching to a different environment while sampling information. Learning progress (left) and time (right) are related to the likelihood of switching environments (expressed in hazard). Hazard is reported in arbitary units (a.u.).

We examined whether exploration was driven by learning progress, by novelty seeking, or if it was random. Specifically, we used a generalized additive model with time-varying covariates to predict whether, on each trial, participants stayed in the same environment or switched to a different one. Participants were included as random effect in the model, and the total number of trials that each participant played was added as a covariate. All continuous variables were standardized before the analyses. As displayed in Fig. 3, we found a significant effect of learning progress (*χ*^2^(1) = 8.06, *p* = .005), and time (*χ*^2^(4) = 54.24, *p* < .001), while novelty did not show a significant effect (*χ*^2^(1) = 2.86, *p* = .09). Since learning progress has a linear relationship with the likelihood of participants' decision to stop sampling an environment, we ran an additional model where learning progress was included as a parametric regressor, allowing us to compute the effect size (*z* = −2.84, *β* = −0.13, *p* = .005, e^β^ = 0.88, 95% CI = [0.802, 0.961]). The total number of trials played by each participant did not affect the results (*z* = 1.33, *β* = 0.006, *p* = .182, e^β^ = 1.01, 95% CI = [0.997, 1.015]).

### 3.2 Environment selection

**Fig. 4:**
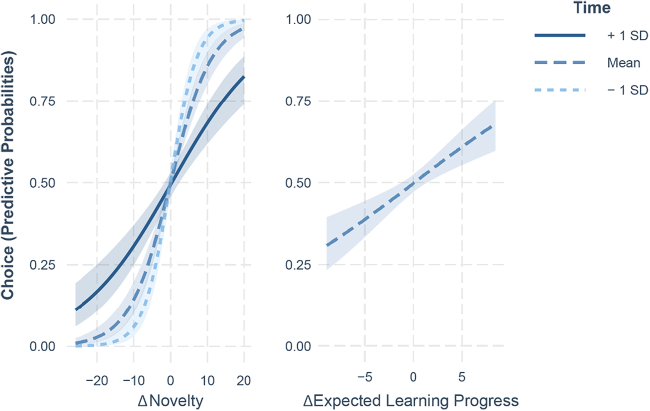
Analysis of the probability of choosing which environment to sample next. The choice was influenced by the difference in novelty between the two competing environments, as well as by the difference in expected learning progress that they offered. Across time, the effect of novelty diminished.

We tested whether participants selected the next environment to sample based on the learning progress that could be expected, its perceptual novelty or simply by chance. Since the expected learning progress can be computed only if participants had already interacted with the environments, the first switch to each environment was excluded from analysis. We fitted a generalized linear model (GLM) on binomial data with differential learning progress and differential novelty as predictors, controlling for their interaction with time. Participants were included as a random factor. As displayed in Fig. 4, the results show that both perceptual novelty (*z* = 6.08, *β* = 0.87, *p* < .001, e^β^ = 2.39, 95% CI = [1.81, 3.19]) and expected learning progress (*z* = 2.84, *β* = 0.24, *p* = .005, e^β^ = 1.27, 95% CI = [1.07, 1.51]) had a positive correlation with participants' choices. The interaction between novelty and time was significant (*z* = −4.12, *β* = −0.49, *p* < .001, e^β^ = 0.61, 95% CI = [0.49, 0.77]), indicating that as time passed, reliance on novelty significantly decreased. The interaction between expected learning progress and time was not significant (*z* = −1.83, *β* = −0.16, *p* = .067, e^β^ = 0.85, 95% CI = [0.71, 1.01]).

### 3.3 Objective performance and subjective experience

**Fig. 5:**
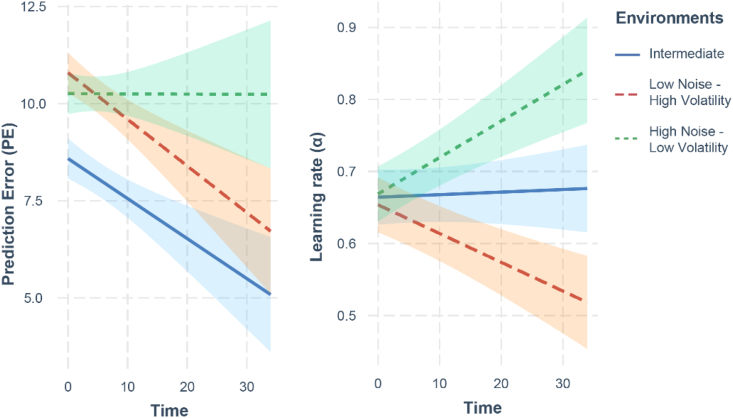
Analysis of how prediction errors and learning rates change over time.

Finally, we tested whether participants were more successful in minimizing prediction error in noisy, volatile, or intermediate environments, and which environment they preferentially engaged with. To test participants' performance, we used a GLM with the trial-by-trial level of prediction error as dependent variable, the three kinds of environments and time as independent variables, and participants as a random factor. We found a significant decrease in prediction errors across time for the intermediate environment (*z* = −2,67, *β* = −0.01, SE = 0.004, *p* = .008, 95% CI = [−0.018, −0.003]) and the high-volatility environment (*z* = −2.87, *β* = 0.01, SE = 0.004, *p* = .004, 95% CI = [−0.020, −0.004]), but not for the high-noise environment (*z* = −0.01, *β* = 0.0001, SE = 0.005, *p* = .99, 95% CI = [−0.010, 0.010]). This shows that participants improved their performance over time in the intermediate and high-volatility environments, but not in the high-noise environment (Fig. 5).

The high-noise environment was inherently difficult to predict, but participants still adapted their behavior depending on the environment. To show this, we used a GLM with the trial-by-trial estimates of the learning rate alpha as dependent variable, the three kinds of environments and time as independent variables, and participants as a random factor. We found a significant interaction between the type of the environment and time, with post-hoc tests showing an increase of alpha over time for the high-noise environment (*z* = 3.04, *β* = 0.01, SE = 0.005, *p* = .002, 95% CI = [0.01, 0.02]), a stable alpha in the intermediate environment (*z* = 0.27, *β* = 0.001, SE = 0.004, *p* = .79, 95% CI = [−0.01, 0.01]), and a decrease in alpha in the high-volatility environment (*z* = −2.80, *β* = −0.01, SE = 0.004, *p* = .005, 95% CI = [−0.018, −0.002]). This shows that participants adapted their learning depending on the type of uncertainty.

**Fig. 6:**
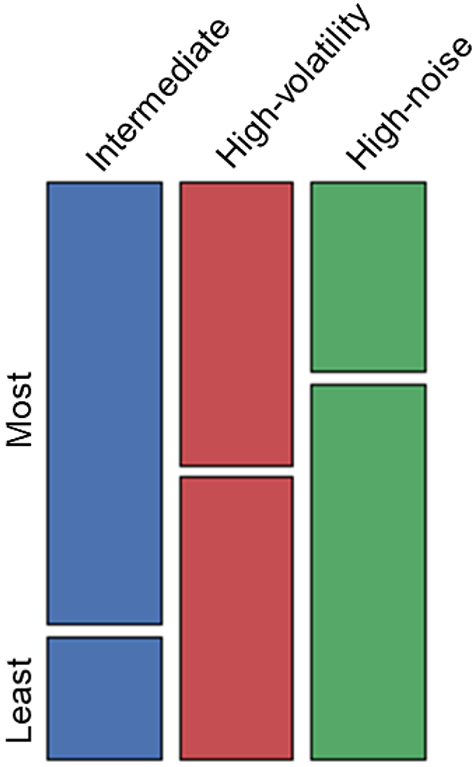
Mosaic plot reporting the explicit reports of which character participants liked the most (‘Most’) and which one they liked the least (‘Least’).

To test whether participants preferred to engage with a specific type of environment, we analyzed their subjective reports of which character they liked the most, and which one they liked the least using a chi-squared test. The pattern of preferences expressed by participants was different from what was expected by chance (*χ*^2^(2) = 9.44, *p* = .009). As illustrated in Fig. 6, participants showed a preference for the intermediate environment.

## 4 Discussion

We investigated what cognitive processes underlie exploration in unknown environments that are devoid of extrinsic rewards. We found that participants' learning progress on the task – measured in terms of reduction in prediction error – affected whether participants kept engaging with the same environment or switched to a different one. Specifically, participants were more likely to keep sampling from the same environment while their learning progress was high, and the likelihood of moving to a different environment increased as the learning progress decreased. This is direct evidence that exploration in humans is driven by the maximization of learning progress.

Once participants had decided to explore a new environment, their choice of which environment to explore next was based on both the perceptual novelty and the expected learning progress associated with an environment. This extends previous work (Wilson, Geana, White, Ludvig, & Cohen, 2014; Zajkowski, Kossut, & Wilson, 2017), which finds multiple coexisting types of exploratory behaviors, revealing the additional, unique contribution of learning progress to exploration in unknown environments. Our definition of expected learning progress builds on existing work on hierarchical reinforcement learning (Wittmann et al., 2016), in which algorithms do not only weight prediction errors depending on the learning rate, but use the same learning rate to compute the prediction error expected in the future. By subtracting expected prediction error from the current prediction error, a new measure of learning progress emerges naturally from reinforcement learning systems. Hence, curiosity can be operationalized within the reinforcement learning framework (see Murayama, FitzGibbon, & Sakaki, 2019), but at the same time it is clearly distinct from the concept of reward. This opens up the possibility of studying intrinsic motivation (i.e., curiosity), extrinsic motivation (i.e., external rewards) and their interaction in a unitary framework.

Our results are in line with the learning progress theory (Oudeyer, 2007), which proposes that optimal learning can be achieved by focusing on activities that allow learning to proceed at a faster pace. This theory is widely used for artificial agents (Matusch, Ba, & Hafner, 2020), but evidence on whether humans use learning progress to structure their exploration has been inconsistent to date (Baranes et al., 2014; Ten, Kaushik, Oudeyer, & Gottlieb, 2021). We showed that learning progress affects both the current engagement with the task, and what task will be chosen in the future. Moreover, the current results inform the predictive coding framework (Clark, 2013) about the specific mechanisms that underlie uncertainty reduction. Predictive coding holds that organisms aim to minimize uncertainty, and our findings offer a mechanistic explanation of how this might be achieved. If agents are driven by a learning-maximization effort, this will also lead them to indirectly minimize environmental unpredictability.

Finally, we investigated how humans respond to environments with different kinds of uncertainty. One of the environments that participants could explore was very noisy, but more stable than the others; another environment was very low in noise, but highly volatile; finally, a third environment had intermediate levels of noise and volatility. We found that participants were able to minimize prediction error in the intermediate and high-volatility environments, but failed to do so in the high-noise environment. However, they adapted their learning rate differentially for each environment, increasing it when the noise was too high, and decreasing it when it was low. This supports the idea that human exploration is flexible across different kinds of environmental uncertainty. In line with a decreased performance in the high-noise environment, participants' explicit reports on which character they liked indicate that they liked the high-noise environment the least. This favors the idea that, even if humans can function well under uncertainty, they prefer avoiding uncertainty when too extreme.

Future work should integrate the cognitive aspects of information seeking with the motivational and affective ones (see for example, Vogl, Pekrun, Murayama, & Loderer, 2020). Recent work showed that uncertainty has a positive value during explorative periods, but negative value during exploitation (Trudel et al., 2020). Moreover, Vellani, De Vries, Gaule, and Sharot (2020) found direct evidence that the level of dopamine, which is a neuromodulator implicated in reward seeking, influences information seeking too, possibly by changing the affective value that is given to information. Whether learning progress itself activates reward circuits in a similar way is still unknown.

Finally, the current study offers a new way of studying exploratory strategies across development. Recent work shows that infants allocate their attention depending on the learning progress offered by environmental stimuli (Poli et al., 2020) and that curiosity triggers their learning and memory retention (Chen, Westermann, & Twomey, 2021). However, we still know little about the active ways that infants use to self-structure their information search (Bazhydai, Westermann, & Parise, 2020). Moreover, young children's exploration-exploitation trade-off is more biased towards exploration compared to adults, and as a consequence they explore more eagerly than adults, but obtain lower rewards (Schulz, Wu, Ruggeri, & Meder, 2019). These differences might originate from a developmental change in the mechanisms underlying curiosity-driven learning, and the current paradigm is suitable for exploring these issues further.

## 5 Conclusions

A growing number of studies showed that artificial agents learn more efficiently and more robustly when they are endowed with an intrinsic reward for learning progress (Pathak et al., 2017). Such a curiosity-driven learning strategy is especially effective when the environment is unknown and devoid of explicit rewards. However, whether humans rely on similar mechanisms when foraging for information in unknown environments was still debated. In the present study, we show that they do. We let participants freely explore different environments that contained learnable (yet noisy) sequences of events. We showed that participants were more likely to stay in the same environment and kept sampling information from it when the learning progress they were making was higher. Moreover, their decision on what environment to sample next was influenced by how much learning progress they expected to make in the chosen environment, and by its perceptual novelty. In conclusion, by use of a novel task and computational modeling, the present study offers new insights on the cognitive strategy that guides human exploration in unknown environments.

## CRediT authorship contribution statement

**Francesco Poli:** Conceptualization, Methodology, Formal analysis, Investigation, Writing – original draft. **Marlene Meyer:** Conceptualization, Methodology, Supervision, Writing – review & editing. **Rogier Mars:** Conceptualization, Methodology, Supervision, Writing – review & editing. **Sabine Hunnius:** Conceptualization, Methodology, Supervision, Writing – review & editing.
